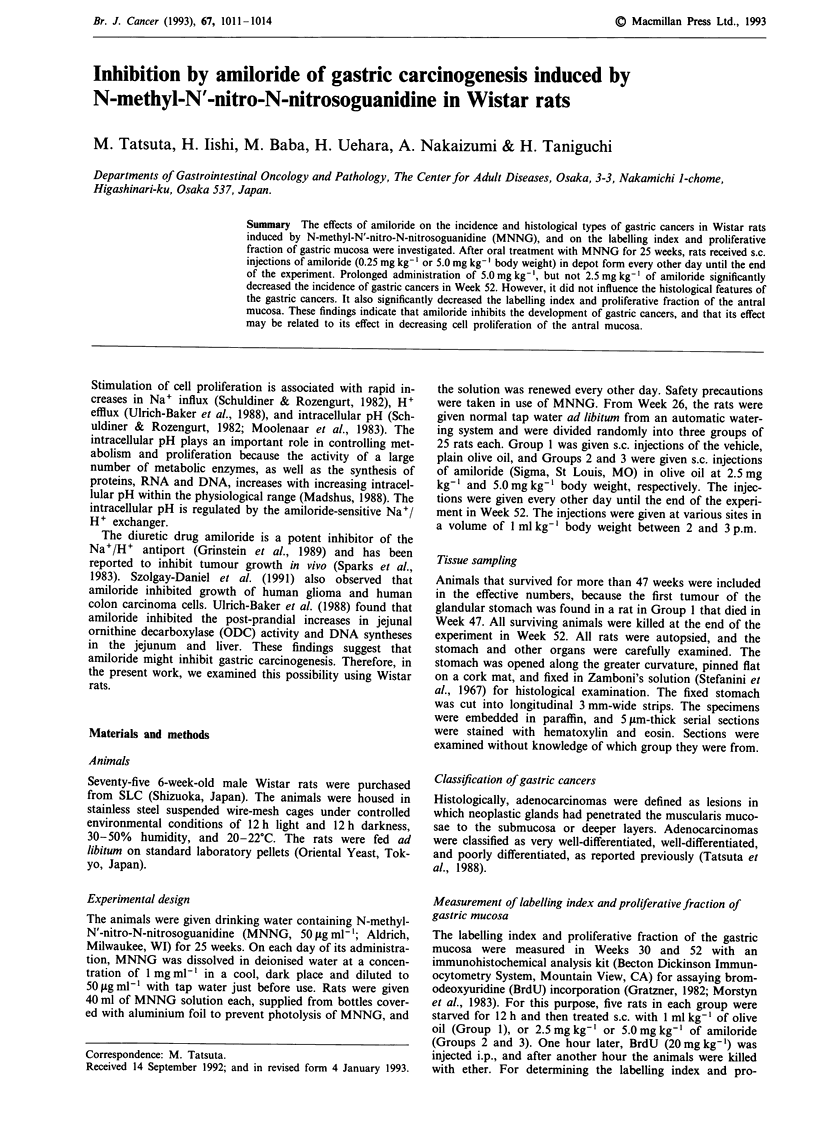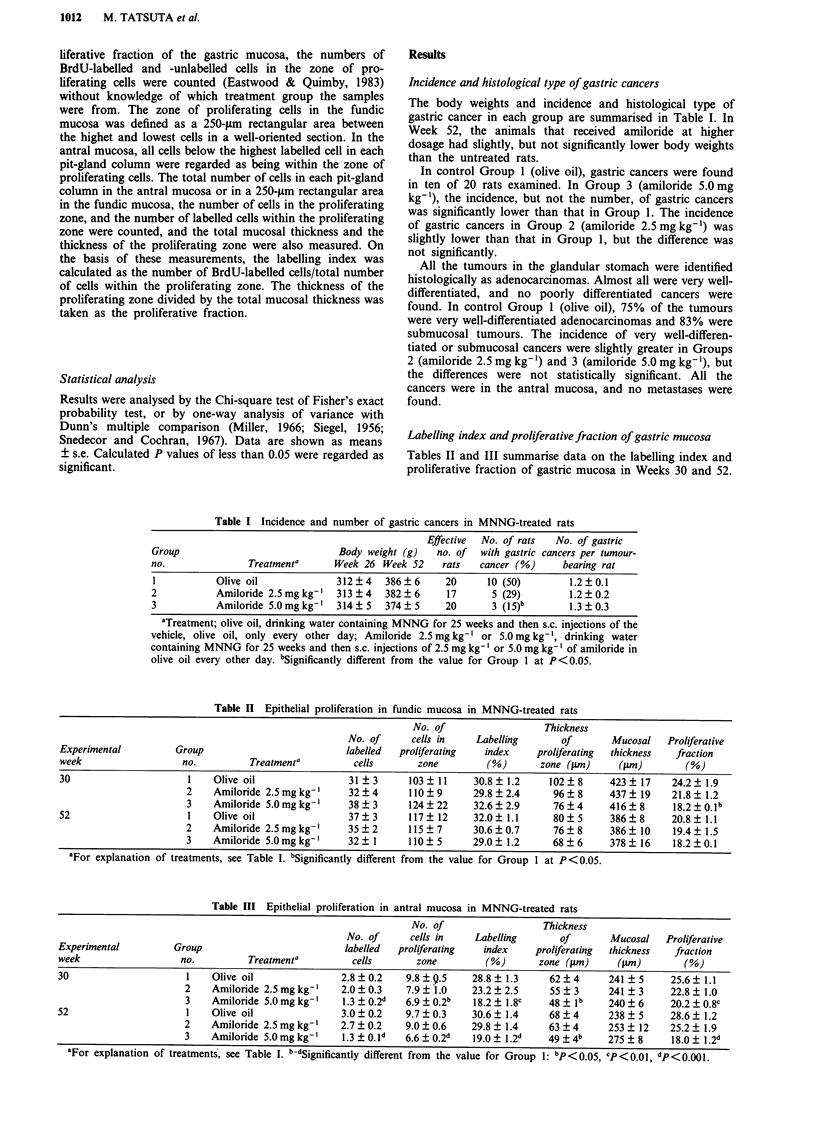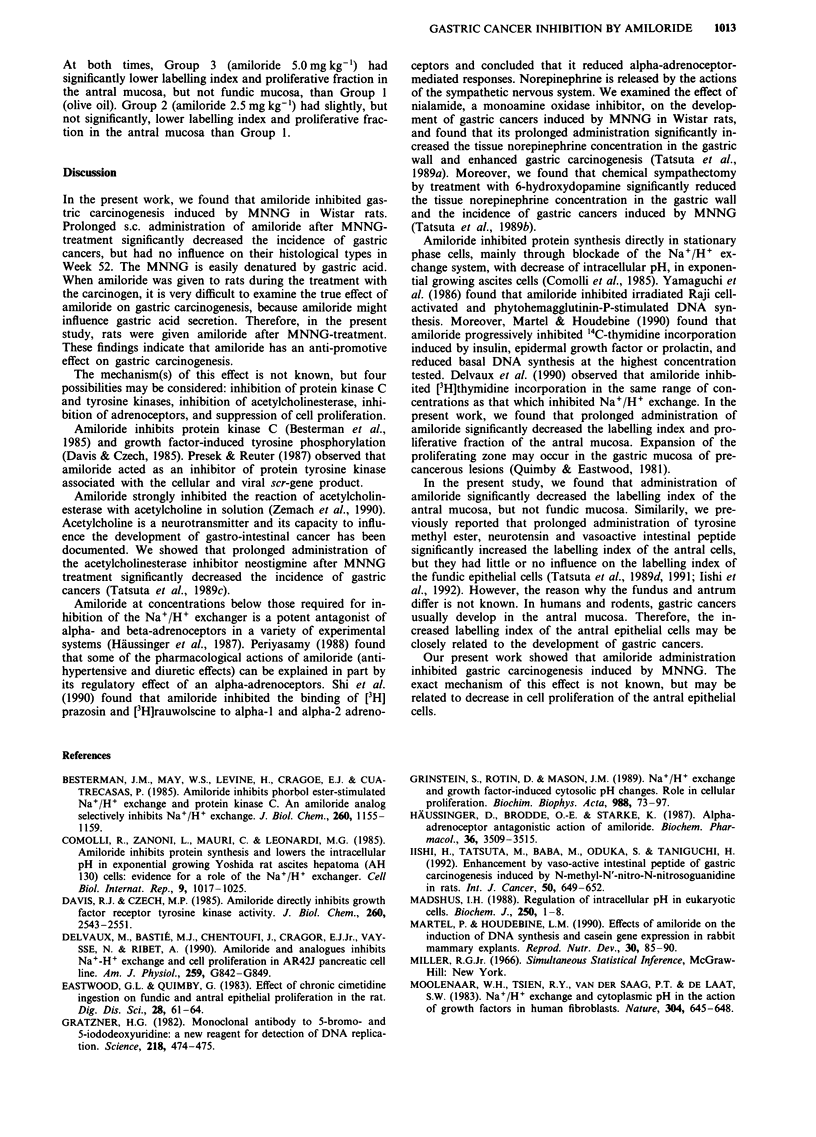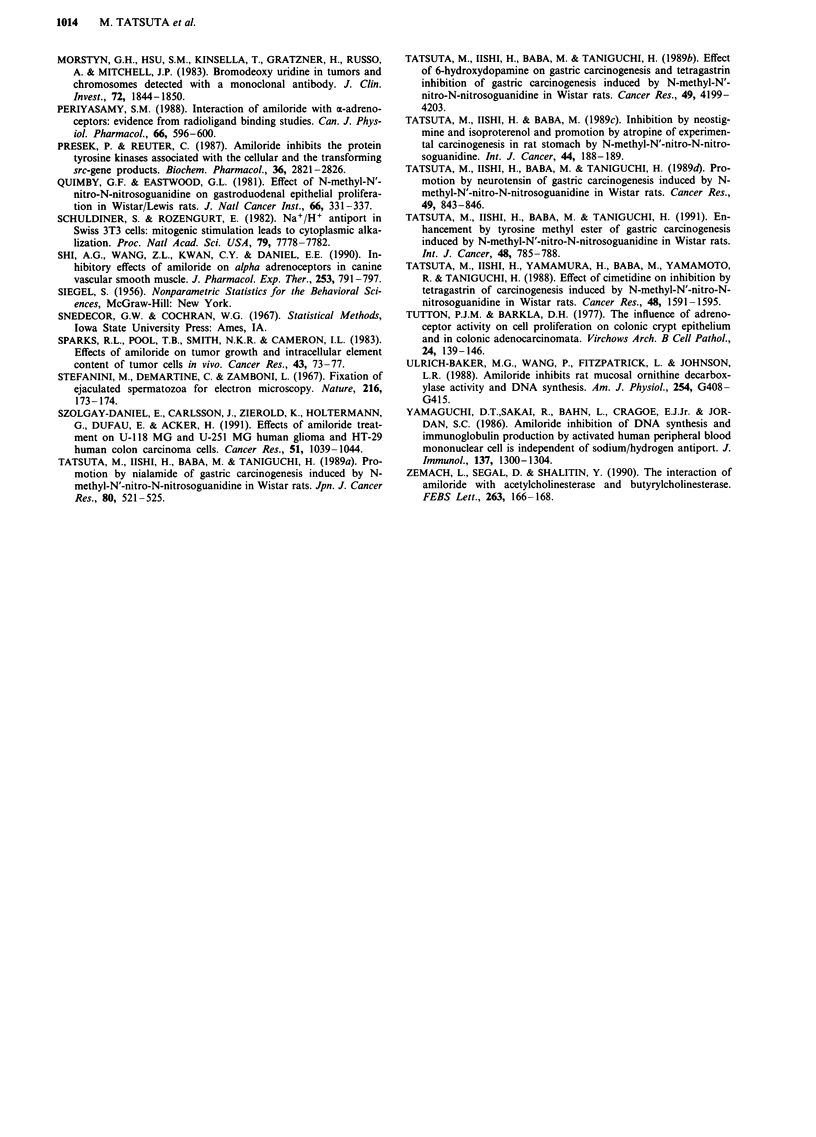# Inhibition by amiloride of gastric carcinogenesis induced by N-methyl-N'-nitro-N-nitrosoguanidine in Wistar rats.

**DOI:** 10.1038/bjc.1993.185

**Published:** 1993-05

**Authors:** M. Tatsuta, H. Iishi, M. Baba, H. Uehara, A. Nakaizumi, H. Taniguchi

**Affiliations:** Department of Gastrointestinal Oncology, Center for Adult Diseases, Osaka, Japan.

## Abstract

The effects of amiloride on the incidence and histological types of gastric cancers in Wistar rats induced by N-methyl-N'-nitro-N-nitrosoguanidine (MNNG), and on the labelling index and proliferative fraction of gastric mucosa were investigated. After oral treatment with MNNG for 25 weeks, rats received s.c. injections of amiloride (0.25 mg kg-1 or 5.0 mg kg-1 body weight) in depot form every other day until the end of the experiment. Prolonged administration of 5.0 mg kg-1, but not 2.5 mg kg-1 of amiloride significantly decreased the incidence of gastric cancers in Week 52. However, it did not influence the histological features of the gastric cancers. It also significantly decreased the labelling index and proliferative fraction of the antral mucosa. These findings indicate that amiloride inhibits the development of gastric cancers, and that its effect may be related to its effect in decreasing cell proliferation of the antral mucosa.


*Br. J. Cancer* (1993), **67**, 1011–1014 © Macmillan Press Ltd., 1993

### Inhibition by amiloride of gastric carcinogenesis induced by N-methyl-N′-nitro-N-nitrosoguanidine in Wistar rats

M. Tatsuta, H. Iishi, M. Baba, H. Uehara, A. Nakaizumi & H. Taniguchi

*Departments of Gastrointestinal Oncology and Pathology, The Center for Adult Diseases, Osaka, 3-3, Nakamichi 1-chome, Higashinari-ku, Osaka 537, Japan.*

**Summary** The effects of amiloride on the incidence and histological types of gastric cancers in Wistar rats induced by N-methyl-N′-nitro-N-nitrosoguanidine (MNNG), and on the labelling index and proliferative fraction of gastric mucosa were investigated. After oral treatment with MNNG for 25 weeks, rats received s.c. injections of amiloride (0.25 mg $kg^{-1}$ or 5.0 mg $kg^{-1}$ body weight) in depot form every other day until the end of the experiment. Prolonged administration of 5.0 mg $kg^{-1}$, but not 2.5 mg $kg^{-1}$ of amiloride significantly decreased the incidence of gastric cancers in Week 52. However, it did not influence the histological features of the gastric cancers. It also significantly decreased the labelling index and proliferative fraction of the antral mucosa. These findings indicate that amiloride inhibits the development of gastric cancers, and that its effect may be related to its effect in decreasing cell proliferation of the antral mucosa.

Stimulation of cell proliferation is associated with rapid increases in $Na^+$ influx (Schuldiner & Rozengurt, 1982), $H^+$ efflux (Ulrich-Baker *et al.*, 1988), and intracellular pH (Schuldiner & Rozengurt, 1982; Moolenaar *et al.*, 1983). The intracellular pH plays an important role in controlling metabolism and proliferation because the activity of a large number of metabolic enzymes, as well as the synthesis of proteins, RNA and DNA, increases with increasing intracellular pH within the physiological range (Madshus, 1988). The intracellular pH is regulated by the amiloride-sensitive $Na^+$/$H^+$ exchanger.

The diuretic drug amiloride is a potent inhibitor of the $Na^+/H^+$ antiport (Grinstein *et al.*, 1989) and has been reported to inhibit tumour growth *in vivo* (Sparks *et al.*, 1983). Szolgay-Daniel *et al.* (1991) also observed that amiloride inhibited growth of human glioma and human colon carcinoma cells. Ulrich-Baker *et al.* (1988) found that amiloride inhibited the post-prandial increases in jejunal ornithine decarboxylase (ODC) activity and DNA syntheses in the jejunum and liver. These findings suggest that amiloride might inhibit gastric carcinogenesis. Therefore, in the present work, we examined this possibility using Wistar rats.

### Materials and methods

### *Animals*

Seventy-five 6-week-old male Wistar rats were purchased from SLC (Shizuoka, Japan). The animals were housed in stainless steel suspended wire-mesh cages under controlled environmental conditions of 12 h light and 12 h darkness, 30–50% humidity, and 20–22°C. The rats were fed *ad libitum* on standard laboratory pellets (Oriental Yeast, Tokyo, Japan).

### *Experimental design*

The animals were given drinking water containing N-methyl-N′-nitro-N-nitrosoguanidine (MNNG, 50 μg $ml^{-1}$; Aldrich, Milwaukee, WI) for 25 weeks. On each day of its administration, MNNG was dissolved in deionised water at a concentration of 1 mg $ml^{-1}$ in a cool, dark place and diluted to 50 μg $ml^{-1}$ with tap water just before use. Rats were given 40 ml of MNNG solution each, supplied from bottles covered with aluminium foil to prevent photolysis of MNNG, and the solution was renewed every other day. Safety precautions were taken in use of MNNG. From Week 26, the rats were given normal tap water *ad libitum* from an automatic watering system and were divided randomly into three groups of 25 rats each. Group 1 was given s.c. injections of the vehicle, plain olive oil, and Groups 2 and 3 were given s.c. injections of amiloride (Sigma, St Louis, MO) in olive oil at 2.5 mg $kg^{-1}$ and 5.0 mg $kg^{-1}$ body weight, respectively. The injections were given every other day until the end of the experiment in Week 52. The injections were given at various sites in a volume of 1 ml $kg^{-1}$ body weight between 2 and 3 p.m.

### *Tissue sampling*

Animals that survived for more than 47 weeks were included in the effective numbers, because the first tumour of the glandular stomach was found in a rat in Group 1 that died in Week 47. All surviving animals were killed at the end of the experiment in Week 52. All rats were autopsied, and the stomach and other organs were carefully examined. The stomach was opened along the greater curvature, pinned flat on a cork mat, and fixed in Zamboni's solution (Stefanini *et al.*, 1967) for histological examination. The fixed stomach was cut into longitudinal 3 mm-wide strips. The specimens were embedded in paraffin, and 5 μm-thick serial sections were stained with hematoxylin and eosin. Sections were examined without knowledge of which group they were from.

### *Classification of gastric cancers*

Histologically, adenocarcinomas were defined as lesions in which neoplastic glands had penetrated the muscularis mucosae to the submucosa or deeper layers. Adenocarcinomas were classified as very well-differentiated, well-differentiated, and poorly differentiated, as reported previously (Tatsuta *et al.*, 1988).

### *Measurement of labelling index and proliferative fraction of gastric mucosa*

The labelling index and proliferative fraction of the gastric mucosa were measured in Weeks 30 and 52 with an immunohistochemical analysis kit (Becton Dickinson Immunocytometry System, Mountain View, CA) for assaying bromodeoxyuridine (BrdU) incorporation (Gratzner, 1982; Morstyn *et al.*, 1983). For this purpose, five rats in each group were starved for 12 h and then treated s.c. with 1 ml $kg^{-1}$ of olive oil (Group 1), or 2.5 mg $kg^{-1}$ or 5.0 mg $kg^{-1}$ of amiloride (Groups 2 and 3). One hour later, BrdU (20 mg $kg^{-1}$) was injected i.p., and after another hour the animals were killed with ether. For determining the labelling index and pro-

Correspondence: M. Tatsuta.
Received 14 September 1992; and in revised form 4 January 1993.

liferative fraction of the gastric mucosa, the numbers of BrdU-labelled and -unlabelled cells in the zone of proliferating cells were counted (Eastwood & Quimby, 1983) without knowledge of which treatment group the samples were from. The zone of proliferating cells in the fundic mucosa was defined as a 250-μm rectangular area between the highet and lowest cells in a well-oriented section. In the antral mucosa, all cells below the highest labelled cell in each pit-gland column were regarded as being within the zone of proliferating cells. The total number of cells in each pit-gland column in the antral mucosa or in a 250-μm rectangular area in the fundic mucosa, the number of cells in the proliferating zone, and the number of labelled cells within the proliferating zone were counted, and the total mucosal thickness and the thickness of the proliferating zone were also measured. On the basis of these measurements, the labelling index was calculated as the number of BrdU-labelled cells/total number of cells within the proliferating zone. The thickness of the proliferating zone divided by the total mucosal thickness was taken as the proliferative fraction.

*Statistical analysis*

Results were analysed by the Chi-square test of Fisher's exact probability test, or by one-way analysis of variance with Dunn's multiple comparison (Miller, 1966; Siegel, 1956; Snedecor and Cochran, 1967). Data are shown as means ± s.e. Calculated $P$ values of less than 0.05 were regarded as significant.

### Results

*Incidence and histological type of gastric cancers*

The body weights and incidence and histological type of gastric cancer in each group are summarised in Table I. In Week 52, the animals that received amiloride at higher dosage had slightly, but not significantly lower body weights than the untreated rats.

In control Group 1 (olive oil), gastric cancers were found in ten of 20 rats examined. In Group 3 (amiloride 5.0 mg $kg^{-1}$), the incidence, but not the number, of gastric cancers was significantly lower than that in Group 1. The incidence of gastric cancers in Group 2 (amiloride 2.5 mg $kg^{-1}$) was slightly lower than that in Group 1, but the difference was not significantly.

All the tumours in the glandular stomach were identified histologically as adenocarcinomas. Almost all were very well-differentiated, and no poorly differentiated cancers were found. In control Group 1 (olive oil), 75% of the tumours were very well-differentiated adenocarcinomas and 83% were submucosal tumours. The incidence of very well-differentiated or submucosal cancers were slightly greater in Groups 2 (amiloride 2.5 mg $kg^{-1}$) and 3 (amiloride 5.0 mg $kg^{-1}$), but the differences were not statistically significant. All the cancers were in the antral mucosa, and no metastases were found.

*Labelling index and proliferative fraction of gastric mucosa*

Tables II and III summarise data on the labelling index and proliferative fraction of gastric mucosa in Weeks 30 and 52.

**Table I** Incidence and number of gastric cancers in MNNG-treated rats

| *Group no.* | *Treatment*[a] | *Body weight (g) Week 26* | *Week 52* | *Effective no. of rats* | *No. of rats with gastric cancer (%)* | *No. of gastric cancers per tumour-bearing rat* |
|---|---|---|---|---|---|---|
| 1 | Olive oil | 312 ± 4 | 386 ± 6 | 20 | 10 (50) | 1.2 ± 0.1 |
| 2 | Amiloride 2.5 mg $kg^{-1}$ | 313 ± 4 | 382 ± 6 | 17 | 5 (29) | 1.2 ± 0.2 |
| 3 | Amiloride 5.0 mg $kg^{-1}$ | 314 ± 5 | 374 ± 5 | 20 | 3 (15)[b] | 1.3 ± 0.3 |

[a]Treatment; olive oil, drinking water containing MNNG for 25 weeks and then s.c. injections of the vehicle, olive oil, only every other day; Amiloride 2.5 mg $kg^{-1}$ or 5.0 mg $kg^{-1}$, drinking water containing MNNG for 25 weeks and then s.c. injections of 2.5 mg $kg^{-1}$ or 5.0 mg $kg^{-1}$ of amiloride in olive oil every other day. [b]Significantly different from the value for Group 1 at $P<0.05$.

**Table II** Epithelial proliferation in fundic mucosa in MNNG-treated rats

| *Experimental week* | *Group no.* | *Treatment*[a] | *No. of labelled cells* | *No. of cells in proliferating zone* | *Labelling index (%)* | *Thickness of proliferating zone (μm)* | *Mucosal thickness (μm)* | *Proliferative fraction (%)* |
|---|---|---|---|---|---|---|---|---|
| 30 | 1 | Olive oil | 31 ± 3 | 103 ± 11 | 30.8 ± 1.2 | 102 ± 8 | 423 ± 17 | 24.2 ± 1.9 |
| | 2 | Amiloride 2.5 mg $kg^{-1}$ | 32 ± 4 | 110 ± 9 | 29.8 ± 2.4 | 96 ± 8 | 437 ± 19 | 21.8 ± 1.2 |
| | 3 | Amiloride 5.0 mg $kg^{-1}$ | 38 ± 3 | 124 ± 22 | 32.6 ± 2.9 | 76 ± 4 | 416 ± 8 | 18.2 ± 0.1[b] |
| 52 | 1 | Olive oil | 37 ± 3 | 117 ± 12 | 32.0 ± 1.1 | 80 ± 5 | 386 ± 8 | 20.8 ± 1.1 |
| | 2 | Amiloride 2.5 mg $kg^{-1}$ | 35 ± 2 | 115 ± 7 | 30.6 ± 0.7 | 76 ± 8 | 386 ± 10 | 19.4 ± 1.5 |
| | 3 | Amiloride 5.0 mg $kg^{-1}$ | 32 ± 1 | 110 ± 5 | 29.0 ± 1.2 | 68 ± 6 | 378 ± 16 | 18.2 ± 0.1 |

[a]For explanation of treatments, see Table I. [b]Significantly different from the value for Group 1 at $P<0.05$.

**Table III** Epithelial proliferation in antral mucosa in MNNG-treated rats

| *Experimental week* | *Group no.* | *Treatment*[a] | *No. of labelled cells* | *No. of cells in proliferating zone* | *Labelling index (%)* | *Thickness of proliferating zone (μm)* | *Mucosal thickness (μm)* | *Proliferative fraction (%)* |
|---|---|---|---|---|---|---|---|---|
| 30 | 1 | Olive oil | 2.8 ± 0.2 | 9.8 ± 0.5 | 28.8 ± 1.3 | 62 ± 4 | 241 ± 5 | 25.6 ± 1.1 |
| | 2 | Amiloride 2.5 mg $kg^{-1}$ | 2.0 ± 0.3 | 7.9 ± 1.0 | 23.2 ± 2.5 | 55 ± 3 | 241 ± 3 | 22.8 ± 1.0 |
| | 3 | Amiloride 5.0 mg $kg^{-1}$ | 1.3 ± 0.2[d] | 6.9 ± 0.2[b] | 18.2 ± 1.8[c] | 48 ± 1[b] | 240 ± 6 | 20.2 ± 0.8[c] |
| 52 | 1 | Olive oil | 3.0 ± 0.2 | 9.7 ± 0.3 | 30.6 ± 1.4 | 68 ± 4 | 238 ± 5 | 28.6 ± 1.2 |
| | 2 | Amiloride 2.5 mg $kg^{-1}$ | 2.7 ± 0.2 | 9.0 ± 0.6 | 29.8 ± 1.4 | 63 ± 4 | 253 ± 12 | 25.2 ± 1.9 |
| | 3 | Amiloride 5.0 mg $kg^{-1}$ | 1.3 ± 0.1[d] | 6.6 ± 0.2[d] | 19.0 ± 1.2[d] | 49 ± 4[b] | 275 ± 8 | 18.0 ± 1.2[d] |

[a]For explanation of treatments, see Table I. [b-d]Significantly different from the value for Group 1: [b]$P<0.05$, [c]$P<0.01$, [d]$P<0.001$.

At both times, Group 3 (amiloride 5.0 mg $kg^{-1}$) had significantly lower labelling index and proliferative fraction in the antral mucosa, but not fundic mucosa, than Group 1 (olive oil). Group 2 (amiloride 2.5 mg $kg^{-1}$) had slightly, but not significantly, lower labelling index and proliferative fraction in the antral mucosa than Group 1.

### Discussion

In the present work, we found that amiloride inhibited gastric carcinogenesis induced by MNNG in Wistar rats. Prolonged s.c. administration of amiloride after MNNG-treatment significantly decreased the incidence of gastric cancers, but had no influence on their histological types in Week 52. The MNNG is easily denatured by gastric acid. When amiloride was given to rats during the treatment with the carcinogen, it is very difficult to examine the true effect of amiloride on gastric carcinogenesis, because amiloride might influence gastric acid secretion. Therefore, in the present study, rats were given amiloride after MNNG-treatment. These findings indicate that amiloride has an anti-promotive effect on gastric carcinogenesis.

The mechanism(s) of this effect is not known, but four possibilities may be considered: inhibition of protein kinase C and tyrosine kinases, inhibition of acetylcholinesterase, inhibition of adrenoceptors, and suppression of cell proliferation.

Amiloride inhibits protein kinase C (Besterman *et al.*, 1985) and growth factor-induced tyrosine phosphorylation (Davis & Czech, 1985). Presek & Reuter (1987) observed that amiloride acted as an inhibitor of protein tyrosine kinase associated with the cellular and viral *scr*-gene product.

Amiloride strongly inhibited the reaction of acetylcholinesterase with acetylcholine in solution (Zemach *et al.*, 1990). Acetylcholine is a neurotransmitter and its capacity to influence the development of gastro-intestinal cancer has been documented. We showed that prolonged administration of the acetylcholinesterase inhibitor neostigmine after MNNG treatment significantly decreased the incidence of gastric cancers (Tatsuta *et al.*, 1989*c*).

Amiloride at concentrations below those required for inhibition of the $Na^+/H^+$ exchanger is a potent antagonist of alpha- and beta-adrenoceptors in a variety of experimental systems (Häussinger *et al.*, 1987). Periyasamy (1988) found that some of the pharmacological actions of amiloride (antihypertensive and diuretic effects) can be explained in part by its regulatory effect of an alpha-adrenoceptors. Shi *et al.* (1990) found that amiloride inhibited the binding of [$^3$H] prazosin and [$^3$H]rauwolscine to alpha-1 and alpha-2 adrenoceptors and concluded that it reduced alpha-adrenoceptor-mediated responses. Norepinephrine is released by the actions of the sympathetic nervous system. We examined the effect of nialamide, a monoamine oxidase inhibitor, on the development of gastric cancers induced by MNNG in Wistar rats, and found that its prolonged administration significantly increased the tissue norepinephrine concentration in the gastric wall and enhanced gastric carcinogenesis (Tatsuta *et al.*, 1989*a*). Moreover, we found that chemical sympathectomy by treatment with 6-hydroxydopamine significantly reduced the tissue norepinephrine concentration in the gastric wall and the incidence of gastric cancers induced by MNNG (Tatsuta *et al.*, 1989*b*).

Amiloride inhibited protein synthesis directly in stationary phase cells, mainly through blockade of the $Na^+/H^+$ exchange system, with decrease of intracellular pH, in exponential growing ascites cells (Comolli *et al.*, 1985). Yamaguchi *et al.* (1986) found that amiloride inhibited irradiated Raji cell-activated and phytohemagglutinin-P-stimulated DNA synthesis. Moreover, Martel & Houdebine (1990) found that amiloride progressively inhibited $^{14}$C-thymidine incorporation induced by insulin, epidermal growth factor or prolactin, and reduced basal DNA synthesis at the highest concentration tested. Delvaux *et al.* (1990) observed that amiloride inhibited [$^3$H]thymidine incorporation in the same range of concentrations as that which inhibited $Na^+/H^+$ exchange. In the present work, we found that prolonged administration of amiloride significantly decreased the labelling index and proliferative fraction of the antral mucosa. Expansion of the proliferating zone may occur in the gastric mucosa of precancerous lesions (Quimby & Eastwood, 1981).

In the present study, we found that administration of amiloride significantly decreased the labelling index of the antral mucosa, but not fundic mucosa. Similarily, we previously reported that prolonged administration of tyrosine methyl ester, neurotensin and vasoactive intestinal peptide significantly increased the labelling index of the antral cells, but they had little or no influence on the labelling index of the fundic epithelial cells (Tatsuta *et al.*, 1989*d*, 1991; Iishi *et al.*, 1992). However, the reason why the fundus and antrum differ is not known. In humans and rodents, gastric cancers usually develop in the antral mucosa. Therefore, the increased labelling index of the antral epithelial cells may be closely related to the development of gastric cancers.

Our present work showed that amiloride administration inhibited gastric carcinogenesis induced by MNNG. The exact mechanism of this effect is not known, but may be related to decrease in cell proliferation of the antral epithelial cells.

### References

BESTERMAN, J.M., MAY, W.S., LEVINE, H., CRAGOE, E.J. & CUATRECASAS, P. (1985). Amiloride inhibits phorbol ester-stimulated $Na^+/H^+$ exchange and protein kinase C. An amiloride analog selectively inhibits $Na^+/H^+$ exchange. *J. Biol. Chem.*, **260**, 1155–1159.

COMOLLI, R., ZANONI, L., MAURI, C. & LEONARDI, M.G. (1985). Amiloride inhibits protein synthesis and lowers the intracellular pH in exponential growing Yoshida rat ascites hepatoma (AH 130) cells: evidence for a role of the $Na^+/H^+$ exchanger. *Cell Biol. Internat. Rep.*, **9**, 1017–1025.

DAVIS, R.J. & CZECH, M.P. (1985). Amiloride directly inhibits growth factor receptor tyrosine kinase activity. *J. Biol. Chem.*, **260**, 2543–2551.

DELVAUX, M., BASTIÉ, M.J., CHENTOUFI, J., CRAGOR, E.J.Jr., VAYSSE, N. & RIBET, A. (1990). Amiloride and analogues inhibits $Na^+$-$H^+$ exchange and cell proliferation in AR42J pancreatic cell line. *Am. J. Physiol.*, **259**, G842–G849.

EASTWOOD, G.L. & QUIMBY, G. (1983). Effect of chronic cimetidine ingestion on fundic and antral epithelial proliferation in the rat. *Dig. Dis. Sci.*, **28**, 61–64.

GRATZNER, H.G. (1982). Monoclonal antibody to 5-bromo- and 5-iododeoxyuridine: a new reagent for detection of DNA replication. *Science*, **218**, 474–475.

GRINSTEIN, S., ROTIN, D. & MASON, J.M. (1989). $Na^+/H^+$ exchange and growth factor-induced cytosolic pH changes. Role in cellular proliferation. *Biochim. Biophys. Acta*, **988**, 73–97.

HÄUSSINGER, D., BRODDE, O.-E. & STARKE, K. (1987). Alpha-adrenoceptor antagonistic action of amiloride. *Biochem. Pharmacol.*, **36**, 3509–3515.

IISHI, H., TATSUTA, M., BABA, M., ODUKA, S. & TANIGUCHI, H. (1992). Enhancement by vaso-active intestinal peptide of gastric carcinogenesis induced by N-methyl-N′-nitro-N-nitrosoguanidine in rats. *Int. J. Cancer*, **50**, 649–652.

MADSHUS, I.H. (1988). Regulation of intracellular pH in eukaryotic cells. *Biochem. J.*, **250**, 1–8.

MARTEL, P. & HOUDEBINE, L.M. (1990). Effects of amiloride on the induction of DNA synthesis and casein gene expression in rabbit mammary explants. *Reprod. Nutr. Dev.*, **30**, 85–90.

MILLER, R.G.Jr. (1966). *Simultaneous Statistical Inference*, McGraw-Hill: New York.

MOOLENAAR, W.H., TSIEN, R.Y., VAN DER SAAG, P.T. & DE LAAT, S.W. (1983). $Na^+/H^+$ exchange and cytoplasmic pH in the action of growth factors in human fibroblasts. *Nature*, **304**, 645–648.

MORSTYN, G.H., HSU, S.M., KINSELLA, T., GRATZNER, H., RUSSO, A. & MITCHELL, J.P. (1983). Bromodeoxy uridine in tumors and chromosomes detected with a monoclonal antibody. *J. Clin. Invest.*, **72**, 1844-1850.

PERIYASAMY, S.M. (1988). Interaction of amiloride with α-adrenoceptors: evidence from radioligand binding studies. *Can. J. Physiol. Pharmacol.*, **66**, 596-600.

PRESEK, P. & REUTER, C. (1987). Amiloride inhibits the protein tyrosine kinases associated with the cellular and the transforming *src*-gene products. *Biochem. Pharmacol.*, **36**, 2821-2826.

QUIMBY, G.F. & EASTWOOD, G.L. (1981). Effect of N-methyl-N'-nitro-N-nitrosoguanidine on gastroduodenal epithelial proliferation in Wistar/Lewis rats. *J. Natl Cancer Inst.*, **66**, 331-337.

SCHULDINER, S. & ROZENGURT, E. (1982). $Na^+/H^+$ antiport in Swiss 3T3 cells: mitogenic stimulation leads to cytoplasmic alkalization. *Proc. Natl Acad. Sci. USA*, **79**, 7778-7782.

SHI, A.G., WANG, Z.L., KWAN, C.Y. & DANIEL, E.E. (1990). Inhibitory effects of amiloride on *alpha* adrenoceptors in canine vascular smooth muscle. *J. Pharmacol. Exp. Ther.*, **253**, 791-797.

SIEGEL, S. (1956). *Nonparametric Statistics for the Behavioral Sciences*, McGraw-Hill: New York.

SNEDECOR, G.W. & COCHRAN, W.G. (1967). *Statistical Methods*, Iowa State University Press: Ames, IA.

SPARKS, R.L., POOL, T.B., SMITH, N.K.R. & CAMERON, I.L. (1983). Effects of amiloride on tumor growth and intracellular element content of tumor cells *in vivo*. *Cancer Res.*, **43**, 73-77.

STEFANINI, M., DEMARTINE, C. & ZAMBONI, L. (1967). Fixation of ejaculated spermatozoa for electron microscopy. *Nature*, **216**, 173-174.

SZOLGAY-DANIEL, E., CARLSSON, J., ZIEROLD, K., HOLTERMANN, G., DUFAU, E. & ACKER, H. (1991). Effects of amiloride treatment on U-118 MG and U-251 MG human glioma and HT-29 human colon carcinoma cells. *Cancer Res.*, **51**, 1039-1044.

TATSUTA, M., IISHI, H., BABA, M. & TANIGUCHI, H. (1989*a*). Promotion by nialamide of gastric carcinogenesis induced by N-methyl-N'-nitro-N-nitrosoguanidine in Wistar rats. *Jpn. J. Cancer Res.*, **80**, 521-525.

TATSUTA, M., IISHI, H., BABA, M. & TANIGUCHI, H. (1989*b*). Effect of 6-hydroxydopamine on gastric carcinogenesis and tetragastrin inhibition of gastric carcinogenesis induced by N-methyl-N'-nitro-N-nitrosoguanidine in Wistar rats. *Cancer Res.*, **49**, 4199-4203.

TATSUTA, M., IISHI, H. & BABA, M. (1989*c*). Inhibition by neostigmine and isoproterenol and promotion by atropine of experimental carcinogenesis in rat stomach by N-methyl-N'-nitro-N-nitrosoguanidine. *Int. J. Cancer*, **44**, 188-189.

TATSUTA, M., IISHI, H., BABA, M. & TANIGUCHI, H. (1989*d*). Promotion by neurotensin of gastric carcinogenesis induced by N-methyl-N'-nitro-N-nitrosoguanidine in Wistar rats. *Cancer Res.*, **49**, 843-846.

TATSUTA, M., IISHI, H., BABA, M. & TANIGUCHI, H. (1991). Enhancement by tyrosine methyl ester of gastric carcinogenesis induced by N-methyl-N'-nitro-N-nitrosoguanidine in Wistar rats. *Int. J. Cancer*, **48**, 785-788.

TATSUTA, M., IISHI, H., YAMAMURA, H., BABA, M., YAMAMOTO, R. & TANIGUCHI, H. (1988). Effect of cimetidine on inhibition by tetragastrin of carcinogenesis induced by N-methyl-N'-nitro-N-nitrosoguanidine in Wistar rats. *Cancer Res.*, **48**, 1591-1595.

TUTTON, P.J.M. & BARKLA, D.H. (1977). The influence of adrenoceptor activity on cell proliferation on colonic crypt epithelium and in colonic adenocarcinomata. *Virchows Arch. B Cell Pathol.*, **24**, 139-146.

ULRICH-BAKER, M.G., WANG, P., FITZPATRICK, L. & JOHNSON, L.R. (1988). Amiloride inhibits rat mucosal ornithine decarboxylase activity and DNA synthesis. *Am. J. Physiol.*, **254**, G408-G415.

YAMAGUCHI, D.T.,SAKAI, R., BAHN, L., CRAGOE, E.J.Jr. & JORDAN, S.C. (1986). Amiloride inhibition of DNA synthesis and immunoglobulin production by activated human peripheral blood mononuclear cell is independent of sodium/hydrogen antiport. *J. Immunol.*, **137**, 1300-1304.

ZEMACH, L., SEGAL, D. & SHALITIN, Y. (1990). The interaction of amiloride with acetylcholinesterase and butyrylcholinesterase. *FEBS Lett.*, **263**, 166-168.